# Sulforaphane induces cell morphology change and cell apoptosis by activating endoplasmic reticulum stress in glioblastoma

**DOI:** 10.1186/s12885-025-14378-4

**Published:** 2025-07-01

**Authors:** Nan Li, Yan Jiang, Ajun Wang, Tongchao Jiang, Huimin Dai, Chengyu Xia, Tongcui Jiang

**Affiliations:** 1 Department of Neurosurgery, The First Affiliated Hospital of USTC, Division of Life Sciences and Medicine, University of Science and Technology of China, Hefei, China; 2 Department of Biochemistry & Molecular Biology, School of Basic Medical Sciences, Anhui Medical University, Hefei, China; 3 Department of Neurosurgery, the First Affiliated Hospital of Guangzhou Medical University, Guangzhou, China; 4 Robert H. Lurie Comprehensive Cancer Center, Northwestern University Feinberg School of Medicine, Chicago, USA

**Keywords:** Sulforaphane, Endoplasmic reticulum stress, Unfolded protein response, Cleaved Caspase-3, Glioblastoma, In vivo model

## Abstract

**Background:**

Sulforaphane (SFN), a naturally occurring isothiocyanate derived from cruciferous vegetables, has shown promise as a multitargeted therapeutic agent in glioblastoma (GBM). This study aimed to elucidate the role and underlying molecular mechanisms of SFN in regulating GBM progression, particularly through the endoplasmic reticulum stress (ERS) and unfolded protein response (UPR) pathways.

**Methods:**

Primary human glioma cells and established GBM cell lines were treated with various concentrations of SFN. RNA sequencing and qPCR analyses were conducted to identify transcriptional changes associated with the UPR pathway. Western blot and immunofluorescence were used to assess the expression and subcellular localization of key ER stress–related proteins. A CHOP knockdown model was employed to examine the functional role of CHOP in SFN-induced apoptosis. Additionally, normal human astrocytes (HA) were used to evaluate the selectivity of SFN’s cytotoxicity. In vivo validation was performed using an intracranial glioma xenograft mouse model.

**Results:**

SFN significantly induced apoptotic cell death in GBM cells. Mechanistically, SFN activated multiple branches of the UPR, notably increasing the expression and nuclear translocation of ATF4 and CHOP. CHOP knockdown markedly attenuated SFN-induced apoptosis. RNA-seq and KEGG enrichment analyses confirmed the involvement of the ER stress pathway. Treatment with 4-phenylbutyrate (4-PBA) suppressed SFN-induced cytotoxicity, further supporting ER stress–mediated apoptosis. In vivo, SFN reduced tumor burden and upregulated ER stress markers in intracranial tumor tissues. Importantly, SFN had minimal cytotoxic effects on normal astrocytes, suggesting a favorable therapeutic window.

**Conclusions:**

This study demonstrates that SFN induces GBM cell apoptosis via activation of the UPR pathway, particularly through the ATF4–CHOP axis. These findings support the potential of SFN as a promising therapeutic agent for glioblastoma.

**Supplementary Information:**

The online version contains supplementary material available at 10.1186/s12885-025-14378-4.

## Introduction

Glioblastoma (GBM) is the most lethal form of primary brain cancer in adults, with a median survival time of only 15 to 18 months despite aggressive multimodal treatment [1, 13]. GBM tumors exhibit a high degree of invasiveness, making it impossible to completely remove the areas of the brain infiltrated by the tumor without compromising essential neurological functions. The inherent infiltration of tumor cells coupled with the cytotoxic effects of treatment leads to neurological deficits and cognitive decline in patients, thereby diminishing their overall quality of life [21, 25]. Therefore, there is an urgent need for therapeutic strategies that can effectively target GBM while minimizing neurotoxicity. GBM tumors are characterized by the disruption of various regulatory pathways, including those involving the p53, Rb, and receptor tyrosine kinase signaling cascades [6, 7, 26]. Mutations in these pathways enable cells to bypass essential tumor-suppressive mechanisms, including cell-cycle regulation, cellular senescence, and programmed cell death, thereby facilitating tumor progression. Despite extensive research efforts, no novel therapeutic agents have demonstrated significant clinical efficacy against the core oncogenic drivers in GBM [5]. Moreover, most single-agent targeted therapies induce compensatory feedback mechanisms that contribute to rapid development of therapeutic resistance.

Sulforaphane (SFN) is an isothiocyanate derived from cruciferous vegetables, has emerged as a promising compound in cancer research due to its multifaceted biological activities, including antioxidant, anti-inflammatory, and anticancer properties [2, 9, 11]. Like some other traditional Chinese medicinal materials that have anti-glioma effects [10, 29], SFN also possesses the same function. Recent reviews have highlighted SFN’s ability to induce apoptosis and inhibit cell invasion in GBM cells by modulating various molecular pathways, including those involved in endoplasmic reticulum (ER) stress [8, 18]. ER stress plays a crucial role in the progression of many cancers, including glioblastoma, as it can lead to the activation of pro-survival pathways that promote tumor growth and resistance to treatment [8]. Acting as an activator of the nuclear factor erythroid 2-related factor 2 (Nrf2), SFN has garnered attention for its protective roles, such as promoting anticancer activities and countering the detrimental effects of toxic substances [4]. Recent extensive research on SFN, both in vivo and in vitro, has underscored its capacity to regulate oxidative stress and antioxidant levels, influence neuroinflammation, and address a spectrum of biochemical imbalances [3]. Mechanistic studies have revealed that SFN modulates the expression of various tumor suppressor and inflammatory genes by targeting pathways such as HDAC, DNMT, and NF-κB [15]. However, the specific mechanisms by which SFN induces apoptosis in GBM, particularly in the context of ER stress and UPR activation, remain largely undefined.

Therefore, the present study aims to investigate whether SFN can induce ER stress and activate the UPR pathway in both established GBM cell lines and patient-derived primary glioma cells, ultimately leading to apoptosis and suppression of tumor growth.

## Materials and methods

### Patients

We gathered 5 specimens of glioma tissues and collected from the Department of Neurosurgery, First Affiliated Hospital of USTC. The ethics committee at the First Affiliated Hospital of USTC (Anhui Provincial Hospital) granted approval for all protocols involving human specimens. Additionally, the Institutional Review Board of the same hospital also approved all protocols. Informed consent was obtained in writing from all patients involved. The samples were obtained following a strict protocol with informed consent, ensuring sterile collection and immediate storage under appropriate conditions. Pathologists categorized all subjects as either normal controls or glioma of different grades samples. The tumor features and relevant clinical data are presented in Supplementary table S1.

### Processing of primary glioma cells

Fresh glioma tissues obtained during surgical resection were immediately placed on dry ice to preserve cell viability. The samples were then transferred to sterile tubes and mechanically dissociated into small fragments under a biosafety cabinet using surgical scissors. Cells were cultured in DMEM/F12 medium (Gibco, USA) supplemented with epidermal growth factor (EGF, 20 ng/ml, Peprotech, USA), basic fibroblast growth factor (bFGF, 20 ng/ml, Peprotech, USA), and B27 supplement (1:50 dilution, Gibco, USA). Cultures were maintained on laminin-coated plates (Corning, USA), and only low-passage cells (passages 2–4) were used for subsequent experiments.

### Cell culture and drug treatment

Cell cultures were established using primary glioma cells, U87, U251 cells and human astrocytes (HA cells) (Procell, Wuhan, China). Primary glioma cells were cultured as described above. The U87 and U251 cells were maintained in in DMEM medium supplemented with 10% FBS and 1% penicillin/streptomycin. Under standard culture conditions of 37 °C in a humidified atmosphere containing 5% CO2. The cells were passaged every 2–3 days using trypsin (Beyotime, C0201) when they reached approximately 90% confluence. For the drug treatment experiments, cells were seeded in 12-well plates (Nest, 701201) and allowed to grow until they reached 70% confluence. U87 and U251 cells were treated with different concentrations of SFN and incubated for various durations. Primary glioma cells pretreated with the ER stress inhibitor 4-PBA (7 mM) for 4 h, followed by incubation with SFN (40 µM) or TM (2.5 µM) for 24 h. After treatment, the cells were either harvested for further analysis.

### Chemicals and reagents

Sulforaphane was acquired from Selleck (Shanghai, China) and reconstituted in dimethyl sulfoxide (DMSO) to a concentration of 5.7 mM, yielding a stock solution. This solution was then preserved at a temperature of -20 °C. U87 and U251 glioblastoma cells were treated with SFN at concentrations of 20 µM, 40 µM, and 60 µM. SFN was diluted to the working concentrations prior to treatment. The final DMSO concentration was kept below 0.1% in all experimental groups. The Cell Counting Kit-8 (CCK-8) assay was purchased from Biosharp (China). The IHC assay kit was purchased from ZSGB-BIO (Beijing, China). 4-PBA, TM (Beyotime, China).

### RNA sequencing

Total RNA was extracted from U251 glioblastoma cells treated with SFN 60 µM for 24 h and vehicle control using TRIzol reagent (Invitrogen, USA), according to the manufacturer’s instructions. RNA quantity and integrity were assessed using a NanoDrop spectrophotometer and Agilent 2100 Bioanalyzer. Library construction and sequencing were performed by TSINGKE Biotech (Beijing, China) using the Illumina NovaSeq 6000 platform with paired-end 150 bp reads. Raw reads were quality-checked using FastQC and trimmed using Trimmomatic. Clean reads were aligned to the human reference genome (GRCh38) using STAR (v2.7.3a). Gene expression quantification was performed with featureCounts (v2.0.1). Differential gene expression analysis was conducted using the DESeq2 R package (v1.34.0), and genes with an adjusted p-value < 0.05 and|log₂ fold change| ≥ 1 were considered significantly differentially expressed.Functional enrichment analysis was performed using the clusterProfiler (v4.2.2) package in R. Gene Ontology (GO) and Kyoto Encyclopedia of Genes and Genomes (KEGG) pathway enrichment were carried out to identify biological processes and pathways associated with SFN treatment. Enrichment results were visualized using dot plots and bar charts. A volcano plot and heatmap of top DEGs were generated using ggplot2 and pheatmap packages, respectively.

### RNA extraction and RT-qPCR

Total RNA extraction from cells was conducted utilizing Trizol reagent (Invitrogen, USA) in accordance with the provided protocol. Subsequent cDNA synthesis was executed with the Evo M-MLV RT Premix for qPCR Kit (Accurate Biotechnology (Human) Co., Ltd). For the quantification of gene expression, real-time qPCR was performed utilizing the SYBR^®^ Green Premix Pro Taq HS qPCR Kit (Accurate Biotechnology (Human) Co., Ltd) on the LightCycler^®^96 platform (Roche). Gene-specific primers, as listed in supplementary table S2, were synthesized by TSINGKE Biotech. The qPCR reaction mixture comprised 10 µL of 2× SYBR Green Pro Taq HS Premix, 100 ng of template DNA, 0.4 µL of each forward and reverse primer, and was adjusted to a final volume of 20 µL with RNase-free water. The cycling conditions were as follows: initial denaturation at 95 °C for 30 s, followed by 45 cycles of 5 s at 95 °C and 30 s at 65 °C, culminating in a melting curve analysis. Glyceraldehyde-3-phosphate dehydrogenase (GAPDH) served as the endogenous control for normalization of Cq values across groups. Relative gene expression levels were then determined using the 2^−△△Ct^ method.

### CCK8 assay

The cell viability was assessed using the CCK8 assay kit (Biosharp, China). Cells were collected and 3 × 10^3^ cells were seeded into each well of 96-well plates. Cell viability was measured after 4 h started from the addition of 10 µL CCK8 reagent to each well. The absorbance value at 450 nm was read by a microplate reader (Thermo Fisher Scientific, USA).

### TUNEL staining

Apoptotic cells were detected using the TUNEL staining kit (Beyotime, China). Experimental procedures were performed according to the user’s instructions. Cells were seeded onto glass slides. After cells attached to slides, the cells were fixed with 4% paraformaldehyde for 30 min and washed using PBS. The samples were permeabilized using 0.1% Triton X-100 for 5 min at room temperature. The TUNEL assay solution was configured according to the instructions and thoroughly mixed. The TUNEL reaction mixture was added to the slides and incubated for 1 h at 37 °C. The slides were then mounted with DAPI for 10 min at room temperature. Apoptotic cell counts were conducted using a fluorescence microscope, with TUNEL-positive cells enumerated in three randomly selected high-magnification (100×) fields per section.

### Western blot analysis

Total protein was extracted from primary glioma cells, U87 and U251 cells using RIPA buffer (Beyotime, China) supplemented with protease inhibitor PMSF (Beyotime, China) or cocktail (Sigma, USA). 5×SDS loading buffer was added, and then samples were metal bathed for ten minutes. Protein was separated by SDS-PAGE (Yazyme, China) and transferred onto PVDF membranes. The membranes were blocked with 5% fat-free milk dissolved in PBST for an hour and incubated overnight with primary antibodies. After washing three time for 10 min respectively, the membranes were incubated with secondary antibodies for an hour at room temperature. The protein bands were visualized using enhanced chemiluminescence reagents (Biosharp, China) and the gray value was calculated using ImageJ software. Antibodies used in this study were listed in the Supplementary table S3.

### Immunohistochemistry

Paraffin-embedded tissue sections from clinical glioma samples were deparaffinized and rehydrated with progressively decreasing concentrations of ethanol solution. Antigen retrieval was performed by boiling the samples in citrate buffer (ZSGB-BIO, Beijing) for 5 min, 3-minute interval and 3-minutes boiling again. The sections were naturally cooled to room temperature. After washing with PBS for 3 times, the sections were treated with 3% hydrogen peroxide for 10 min. The sections were then blocked with ADB for 30 min at 37℃ and incubated overnight with primary antibodies. After washing with PBS for three times, the sections were incubated with secondary antibodies (ZSGB-BIO, Beijing) for 30 min at 37℃. After washing away secondary antibodies, DAB reagent (ZSGB-BIO, Beijing) was used to produce a positive reaction. Then sections were immersed in xylene for four hours. Sections were sealed using xylene and neutral resin and observed by microscopy. The average optical density value was calculated by ImageJ software. Antibodies used in this study were listed in the Supplementary table S3.

### Immunofluorescence staining

Cells were seeded onto glass slides until cells attached to slides. The cells were fixed with 4% paraformaldehyde and permeabilized with 0.1% Triton X-100. The slides were then blocked with 5% bovine serum albumin for 30 min at 37℃ and incubated with primary antibodies overnight at 4℃. After washing with PBS, the slides were incubated with secondary antibodies (Proteintech, China) for an hour and a half at 37℃ in a light protected environment. The slides were incubated with DAPI and then washed using PBS for more than three times. After that the slides were sealed with anti-fade mounting medium. The results were observed under a fluorescence microscope.

### Statistical analysis

All experiments were performed in triplicate, and results are expressed as mean ± standard error of the mean (SEM). Statistical analyses were conducted using GraphPad Prism 9 software. Comparisons between two groups were made using the unpaired Student’s t-test, while comparisons among three or more groups were analyzed using one-way ANOVA. A p-value < 0.05 was considered statistically significant.

## Results

### Sulforaphane reduces viability and promotes apoptosis of GBM cells

**Fig. 1:**
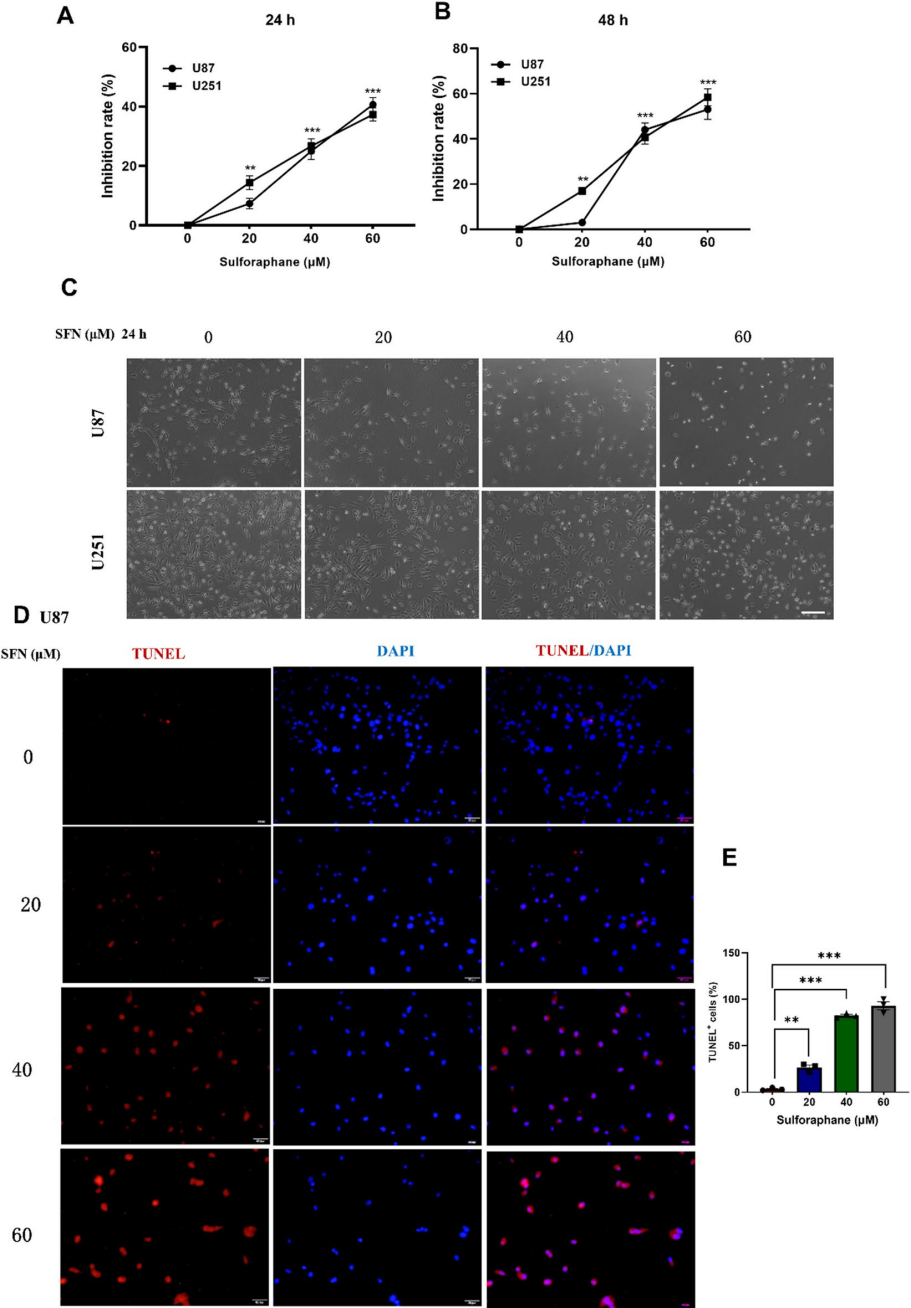
SFN Inhibits Cell Viability and Promotes Cell Apoptosis in Glioma Cells. U87 and U251 cells were incubated with SFN at the indicated concentrations for 24 h (**A**) or 48 h (**B**). Cell viability was determined by CCK8 assay. Cell morphological changes following the indicated treatment for 24 h were examined and photographed, bar: 200 μm (**C**). TUNEL staining of U87 cells treated with SFN at the indicated concentrations for 24 h (**D**). Quantitative analysis of TUNEL-positive U87 cells in **D** (**E**). Statistical values are expressed as the mean ± SEM of three independent experiments. *P < 0.05, **P < 0.01, ***P < 0.001 vs. the control group

To investigate the viability and the morphology effects of sulforaphane (SFN) on U87 and U251 GBM cells, we first analyzed the crucial role of SFN in GBM cell viability by using CCK-8 assay. Treatment with SFN for 24 and 48 h resulted in a higher GBM cells growth inhibition rate (the cell viability of the GBM cells was significantly decreased) in a dose-dependent manner (Fig. 1A, B). In addition, we observed that SFN altered the morphology of the above two lines of GBM cells in a dose-dependent manner. Especially, SFN at 40 µM and 60 µM significantly damaged the normal morphology of the cells, with loss of cellular extensions, membrane blebbing, and detachment from the culture substrate (Fig. 1C). TUNEL assays in U87 cells further revealed that SFN at doses of 20, 40 and 60 µM increased the number of TUNEL-positive cells compared to that in the vehicle control group, the effects of 40 and 60 µM are similar (Fig. 1D, E and Supplementary Fig. 1). To further assess the selectivity and safety of SFN, we additionally performed CCK-8 assays on HA cells in this study. The results showed that SFN, at the concentrations used for glioma treatment, did not significantly affect the viability of normal human astrocytes (Supplementary Fig. 3), suggesting a favorable therapeutic window. These data demonstrate that SFN decreases cell proliferation and promotes cell apoptosis in GBM cells.

### SFN induces ER stress and activates UPR pathway leading to apoptosis in GBM cells

**Fig. 2:**
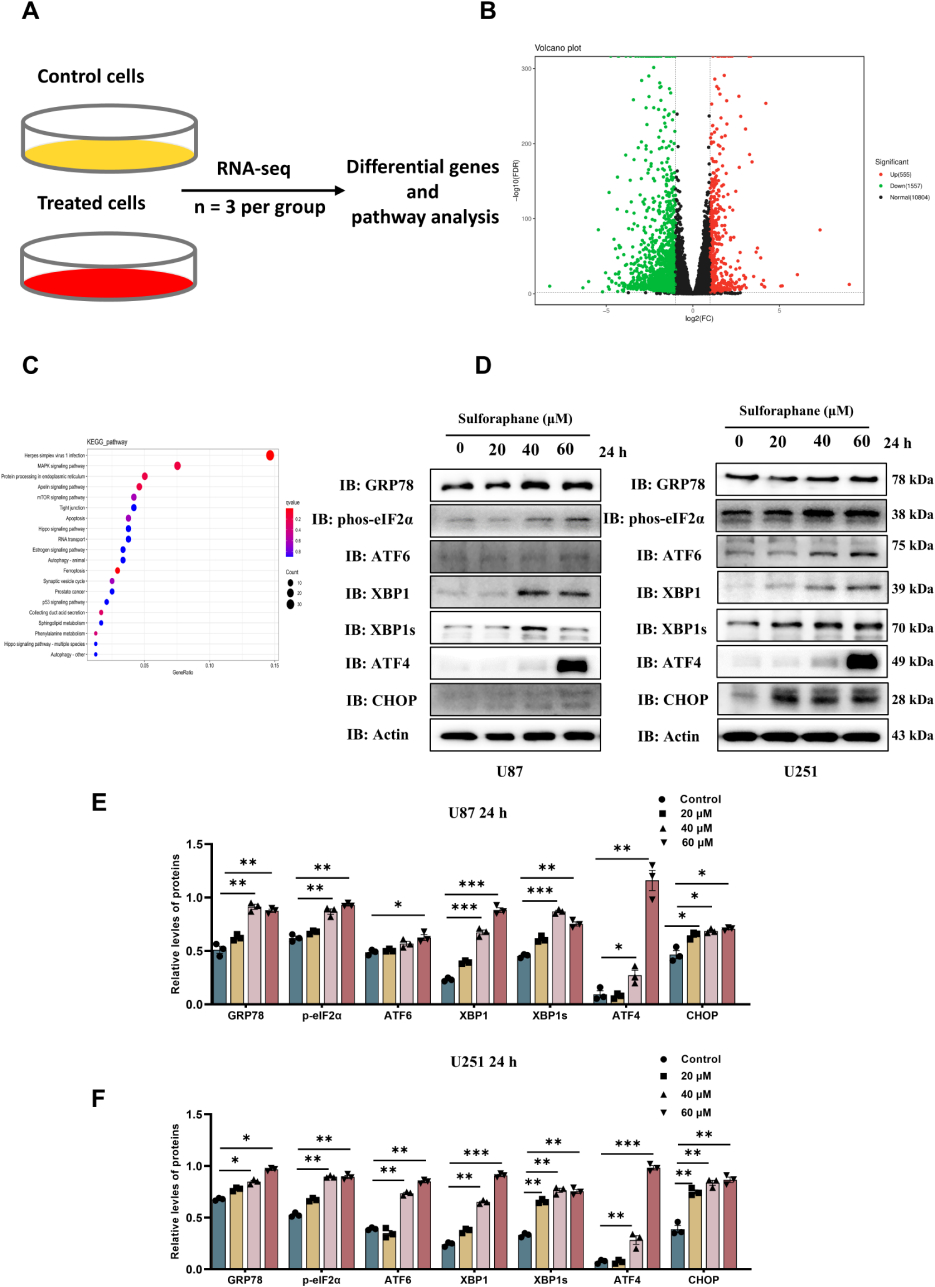
SFN Activates ER Stress Pathways in Glioma Cells. Workflow for RNA-sequencing in two groups (**A**). Volcano plot exhibiting 555 up-regulated and 1557 down-regulated SFN-related genes (**B**). KEGG analysis of different expression gene sets between control and SFN-treated groups (**C**). Representative immunoblots against ER stress-related proteins from U87 and U251 cells treated with SFN (20, 40, and 60 µM) for 24 h (**D**). Quantitative analysis of protein levels in **D** left (**E**) and D right (**F**). Values are expressed as the mean ± SEM of three independent experiments. *P < 0.05, **P < 0.01, ***P < 0.001 vs. the control group

**Fig. 3:**
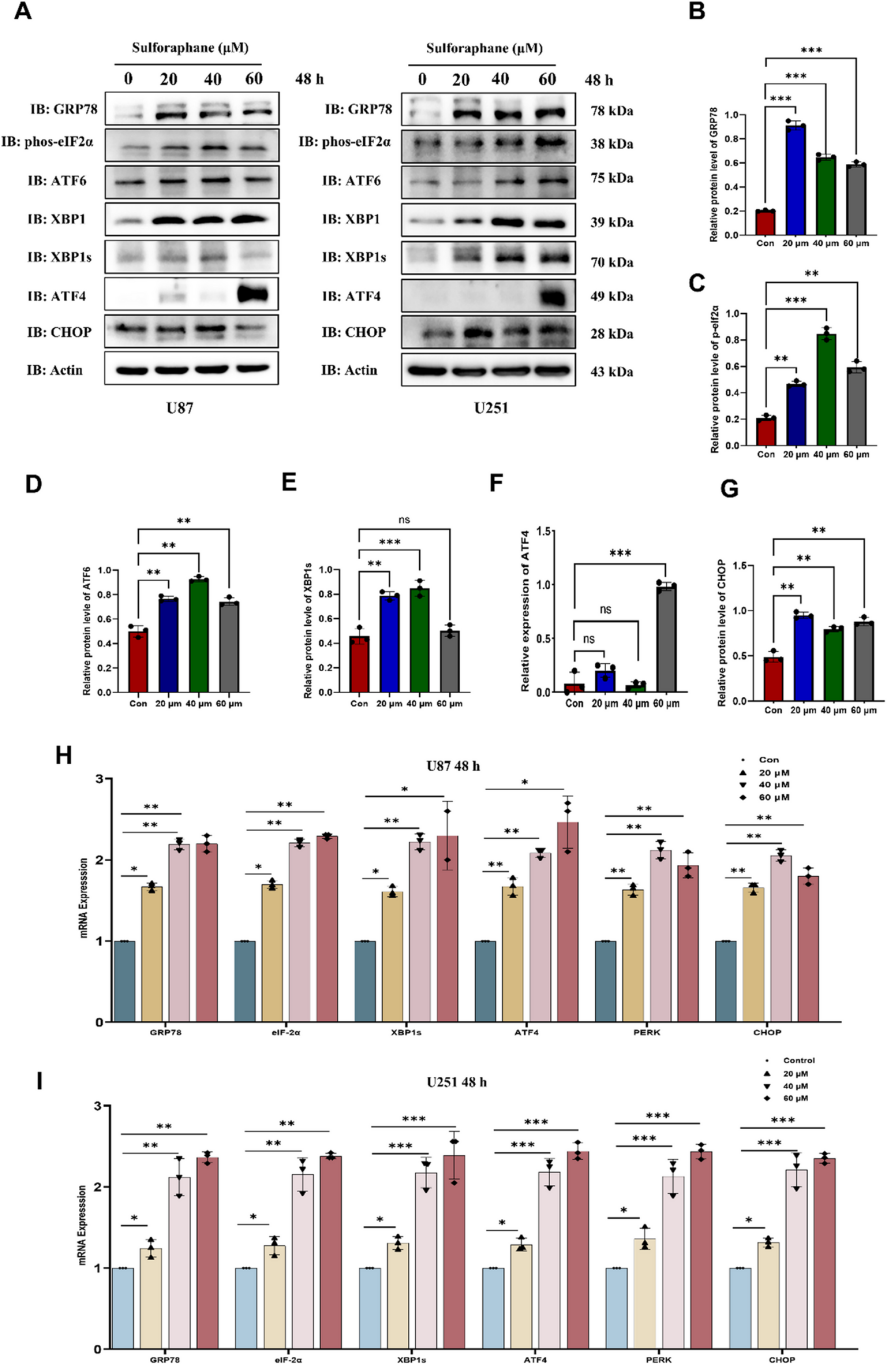
Enhanced ER Stress Response with Increased Stimulation Duration of SFN in Glioma Cells. Representative immunoblots against ER stress-related proteins from U87 cells and U251 cells treated with SFN (20, 40, and 60 µM) for 48 h (**A**). Quantitative analysis of protein levels of GRP78 (**B**), p-eIf2α (**C**), ATF6 (**D**), xBP1s (**E**), ATF4 (**F**) and CHOP (**G**) in A left U87 cells. U87 (**H**) and U251 (**I**) cells were treated with SFN (20, 40, and 60 µM) for 48 h, and the mRNA levels of GRP78, eIf2α, xBP1s, ATF4, PERK and CHOP were assessed by qRT-PCR, with GAPDH serving as a reference gene. Data are presented as the mean ± SEM from three replicate experiments. *P < 0.05, **P < 0.01, ***P < 0.001 vs. the control group

To investigate whether SFN-induced GBM cells apoptosis is related to UPR activation, we conducted RNA-sequencing analysis across two distinct groups (Fig. 2A), revealing a comprehensive view of gene expression changes. The results were graphically represented through a volcano plot (Fig. 2B), which highlighted a significant differential expression pattern: 555 genes were found to be up-regulated, while 1557 genes were down-regulated in relation to SFN. Furthermore, we performed a KEGG analysis to explore the functional implications of these differentially expressed gene sets between the control and SFN-treated groups, shedding light on the molecular mechanisms underlying the observed changes (Fig. 2C). Furthermore, we examined the expression of UPR pathway-related proteins in GBM cells. Glucose regulated protein 78 (GRP78), a key molecule of ER stress, was significant increased after exposure to SFN 24 h at different concentration points in U87 and U251 cells indicated the occurrence of ER stress (Fig. 2D, E and F). We examined key proteins in the three pathways of UPR- phosphorylated eIF2α (p-eIF2α), which can be phosphorylated by the phosphorylated protein kinase R (PKR)-like endoplasmic reticulum kinase (PERK) kinase, XBP1s and ATF6 after SFN treatment. The relative levels of p-eIF2α and ATF6 increased in U87 and U251 cells after SFN exposure compared with those in control cells (Fig. 2D, E and F). Moreover, XBP1s, a key downstream signaling molecule of IRE1 that acts in the IRE1–XBP1 pathway of the UPR, was also increased in U87 cells exposed to SFN (Fig. 2). Furthermore, increased p-eIF2α with a concomitant increase in ATF4 and activated C/EBP homologous protein (CHOP) (Fig. 2D, E and F). Similar results were obtained in U87 and U251 cells treated with SFN in a dose-dependent manner at 48 h (Fig. 3 and supplementary Fig. 2). These findings indicate that SFN induces ER stress in GBM cells.

### SFN changes morphology of GBM cells at a time-dose under ER stress

**Fig. 4:**
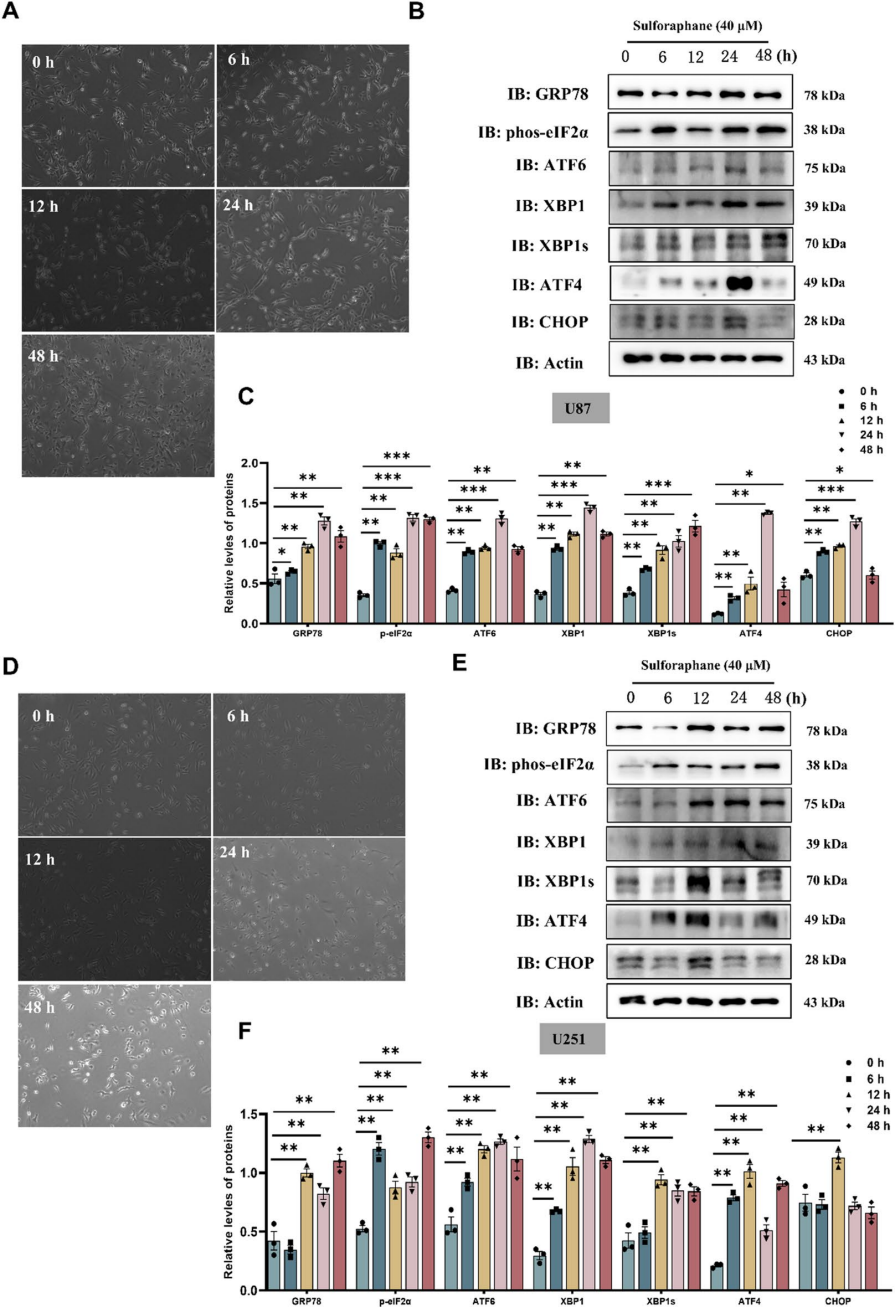
SFN Changes Morphology and Activates ER Stress Pathways in Glioma Cells in Time-dependent Manners. Cell morphological changes following the indicated concentration of 40 µM SFN treated for 6, 12, 24 and 48 h in U87 cells (**A**) and U251 (**D**) cells were examined and photographed. Bar: 100 μm. Representative immunoblots against ER stress-related proteins from U87 cells (**B**) and U251 cells (**E**) treated with SFN 40 µM for 6, 12, 24 and 48 h. Quantitative analysis of protein levels in **B**, **E** (**C**, **F**). Data are presented as the mean ± SEM from three separate experiments.*P < 0.05, **P < 0.01, ***P < 0.001 vs. control

In order to observe the effect of SFN on GBM cell morphology and the change of ER stress-related proteins at different time points. We used U87 and U251 cells treated with 40 µM of SFN for 6, 12, 24, and 48 h, respectively. In both U87 and U251 cells, the morphological changes were more pronounced at 40 μm at 24 h (Fig. 4A, D), while the expression of GRP78, p-eIF2α, ATF6, ATF4, and CHOP was highest at 24 h (Fig. 4B, C, E and F). Collectively, these findings indicate that SFN induces ER stress at a time-dose concomitant with CHOP activation in glioma cells mostly at 24 h.

### SFN-induced nuclear translocation of CHOP and ATF4 in glioma cells

**Fig. 5:**
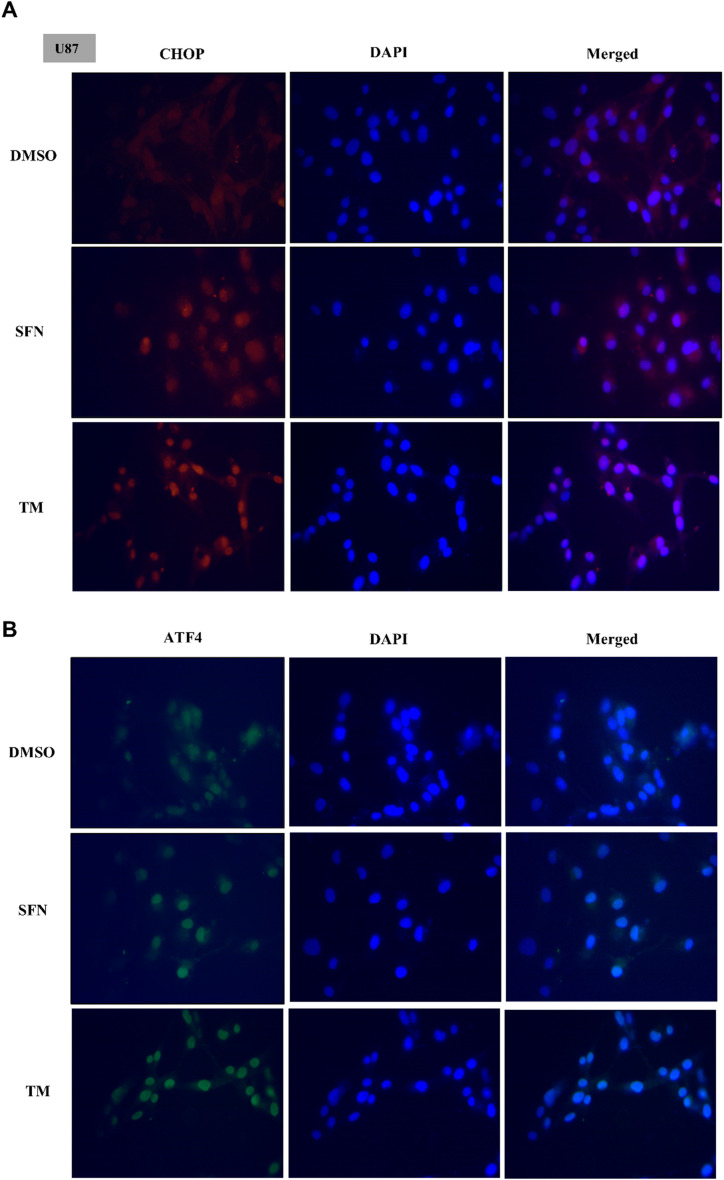
SFN Induces the Nuclear Translocation of CHOP and ATF4 in Glioma Cells. Representative immunofluorescence images depict nuclear translocation of CHOP (**A**) and ATF4 (**B**) in U87 cells following treatment with 40 µM SFN or the ER stress inducer tunicamycin (TM). Scale bar = 100 μm

We explored the link between SFN activation of ER stress and ER stress inducer tunicamycin (TM). The results demonstrated that both SFN 40 µM treatment and TM increased the levels of nuclear translocation of CHOP, a nuclear transcription factor normally sequestered in the cytoplasm (Fig. 5A). To further confirm SFN more potently activated the eIF-2α/ATF4 axis of ER stress, we additionally used immunofluorescence staining and revealed that SFN 40 µM treatment and TM significantly promoted ATF4 nuclear translocation in U87 cells (Fig. 5B). Collectively, these findings indicate that SFN induces ER stress concomitant with CHOP activation in glioma cells.

### 4-PBA mitigates SFN-induced cytotoxicity in glioma cells by inhibiting ER stress

**Fig. 6:**
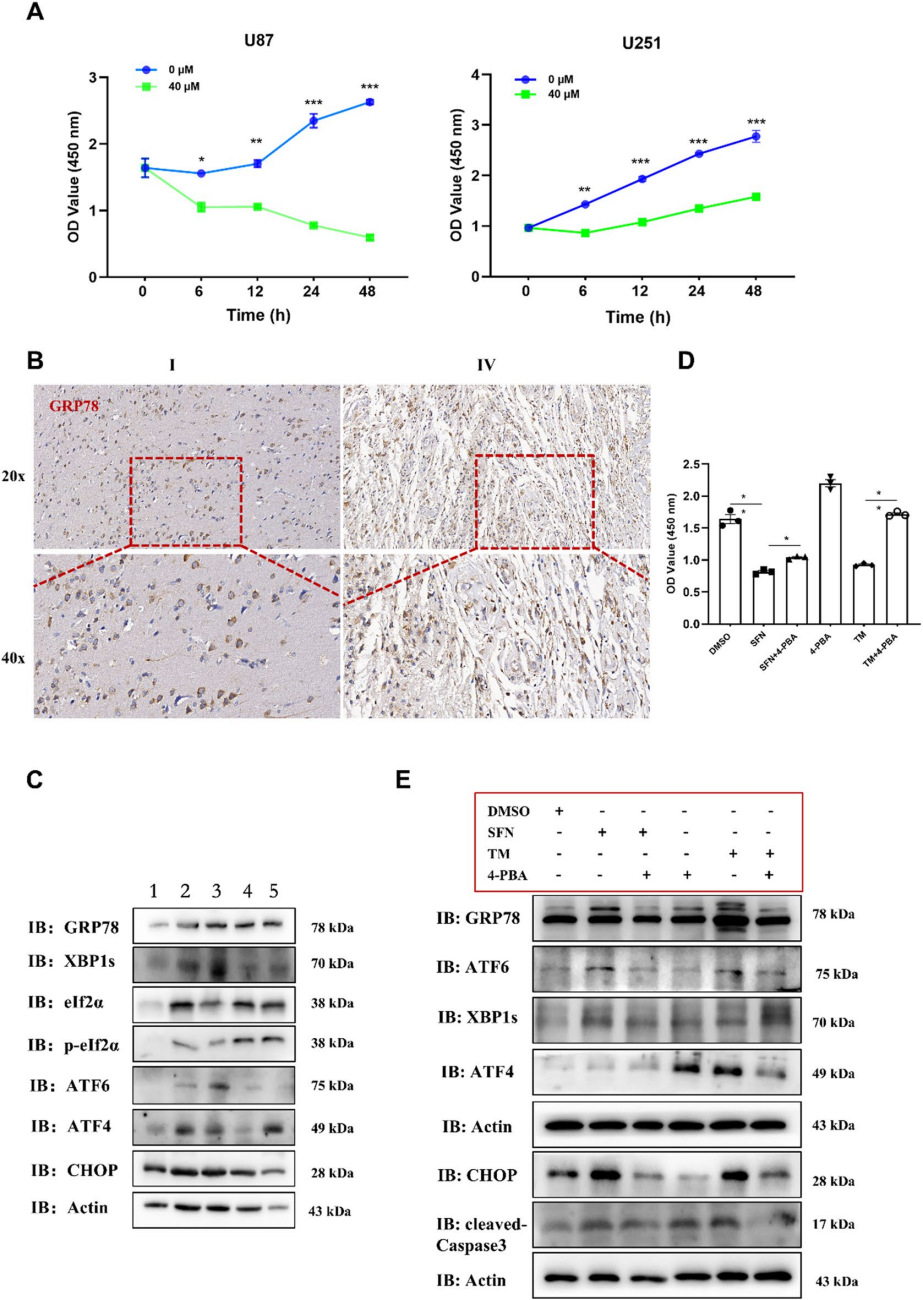
ER Stress Expression in Clinical Samples. U87 and U251 cells were incubated with SFN (40 µM) for 6, 12, 24 and 48 h. The CCK8 assay was used to detect cell viability (**A**). Immunohistochemistry for detecting GRP78 expression in glioma samples (**B**). Western Blot for detecting expression of ER-related markers GRP78, eIf2α, p-eIf2α, ATF6, xBP1s, ATF4 and CHOP in Samples (**C**). Primary glioma cells pretreated with the ER stress inhibitor 4-PBA (7 mM) for 4 h, followed by incubation with SFN (40 µM) or TM (2.5 µM) for 24 h. The CCK8 assay was used to detect cell viability (**D**). The expression levels of the ER stress-related proteins GRP78, ATF6, XBP1s, ATF4, CHOP, and cleaved-caspase3 were determined by WB (**E**). Data are presented as the mean ± SEM from three separate experiments. *P < 0.05, **P < 0.01, ***P < 0.001 vs. control

We further validated the toxic effects of SFN on glioma cells using the CCK8 assay method (Fig. 6A). Additionally, we utilized clinical samples and employed immunohistochemical (Fig. 6B) and Western blot experimental methods to detect the expression of GRP78 and ER stress-related markers. The results revealed that as the grade of glioma increased, the expression levels of ER stress-related markers also increased (Fig. 6C). To determine the involvement of ER stress in SFN-induced apoptosis, we evaluated the efficacy of 4-PBA, a chemical chaperone that alleviates ER stress by decreasing the ER’s misfolded protein load, in reducing the cytotoxic impact of SFN on glioma cells. The results showed that the viability of primary glioma cells was higher when they were pre-treated with 4-PBA before SFN exposure than in cells exposed to SFN alone (Fig. 6D). Additionally, primary glioma cells exposed to SFN exhibited increased levels of CHOP and cleaved caspase-3 compared to the control group (Fig. 6E). To further verify the role of CHOP in SFN-induced apoptosis, we performed siRNA-mediated knockdown of CHOP in U251 cells. CHOP knockdown using specific siRNA significantly attenuated SFN-induced apoptosis, as indicated by reduced cleaved caspase-3 expression.

**Fig. 7:**
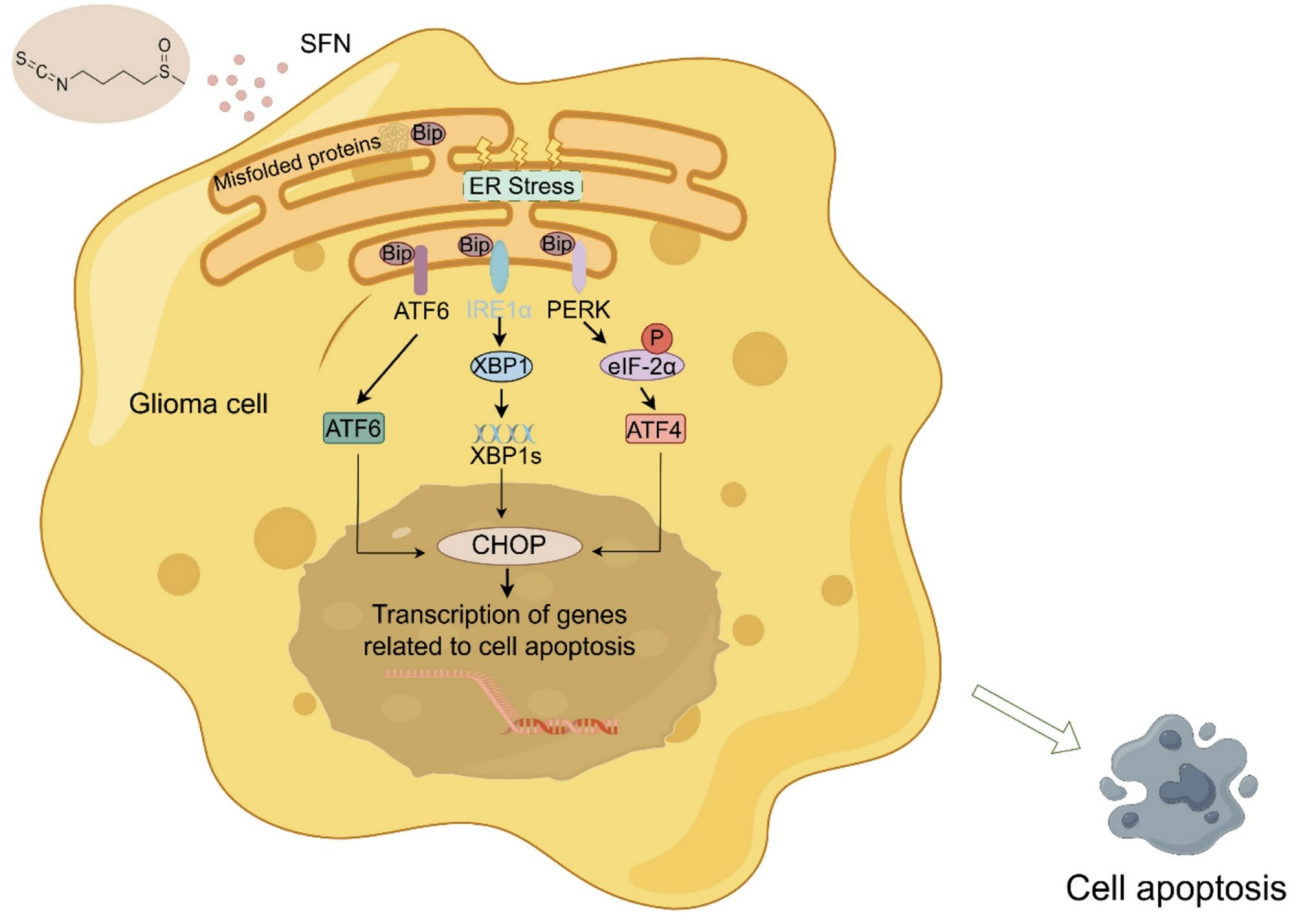
Overview of SFN’s therapeutic potential in glioblastoma

In summary, our study confirmed the cytotoxic effects of SFN on glioma cells through the CCK8 assay, and further analysis using clinical samples and immunohistochemical and Western blot methods indicated a correlation between increased glioma grade and elevated ER stress marker expression. We also demonstrated that 4-PBA, by reducing ER stress, enhanced the survival of primary glioma cells when pretreated before SFN exposure. Moreover, SFN treatment led to a significant upregulation of CHOP and activated caspase-3 in primary glioma cells, highlighting the role of ER stress in SFN-induced apoptosis (Fig. 7, by Figdraw).

## Discussion

Bioactive compounds from natural sources hold substantial promise for treating diseases and are typically viewed as safer alternatives to synthetic agents [30]. sulforaphane (SFN) has gained recognition as an effective agent in lowering the risk of multiple types of cancer, encompassing oral, lung, breast, colon, and prostate [16]. Furthermore, SFN induces apoptosis in cancer cells by increasing mitochondrial permeability, releasing cytochrome C, and modulating proteins like Bcl-2 and caspases, with ROS-dependent pathways activating caspases and cleaving PARP in bladder cancer cells [20]. SFN-Cys may disrupt microtubules, modulate mitophagy, and alter the expression of S100A4 and Claudin-5 to inhibit migration and invasion in glioblastoma (GBM) cells [31]. However, its role in GBM remains largely unexplored. Consequently, our study seeks to evaluate its therapeutic potential in GBM.

Our study provides novel insights into the role of SFN in GBM progression by elucidating its effects on endoplasmic reticulum (ER) stress and the unfolded protein response (UPR) pathway. We found that SFN treatment significantly increased apoptotic cell death in primary glioma cells, which is consistent with previous studies suggesting its potential as a multitargeted therapeutic agent in cancer treatment. Notably, our results demonstrate that SFN induces apoptosis by specifically activating the ER stress pathway, as evidenced by the upregulation of key UPR mediators such as activating transcription factor 4 (ATF4) and CCAAT/enhancer-binding protein homologous protein (CHOP). This finding aligns with the growing body of evidence highlighting the importance of ER stress in modulating apoptosis and cell survival in cancer cells. Our study extends these observations to GBM, providing a compelling rationale for further investigation into SFN’s therapeutic potential in this aggressive form of brain cancer. GRP78 (also known as BiP) is a central regulator of the ER stress response and serves as a marker of UPR activation in cancer therapy [12]. In our study, GRP78 expression was significantly upregulated following SFN treatment, suggesting that SFN induces ER stress in glioblastoma cells. This upregulation is part of the adaptive response to ER stress and precedes the activation of pro-apoptotic factors such as ATF4 and CHOP. Therefore, GRP78 appears to play an upstream regulatory role in SFN-induced ER stress–mediated apoptosis. We recognize that additional comprehensive animal studies are still needed to further evaluate the pharmacokinetics (PK), blood–brain barrier (BBB) permeability, and long-term therapeutic potential of SFN. Given the anatomical and physiological complexity of the BBB, it is critical to determine whether SFN can achieve therapeutic concentrations in intracranial tumors following systemic administration.

In our experiments, we observed that SFN increases ER stress in GBM cells, leading to enhanced apoptosis. However, we acknowledge that further validation of ER stress–related markers (e.g., GRP78, CHOP) using publicly available patient datasets (e.g., TCGA, CGGA) is warranted to determine their clinical correlation and prognostic value in GBM, and this will be explored in future studies. This effect is mediated through the activation of ER stress pathways that ultimately trigger cell death mechanisms. However, ER stress can also have protective effects. For instance, some studies have shown that ER stress can induce the expression of chaperone proteins, which help in refolding misfolded proteins and restoring homeostasis, thereby preventing apoptosis [22, 23]. Jingfang Granules mitigate OVA-induced allergic rhinitis by modulating the ER stress signaling pathway [28]. SFN reversed the effects of bisphenol A (BPA) by alleviating ER stress, which BPA had activated to promote hepatic lipid accumulation [14]. However, on the other hand SFN had an unexpected effect on cardiomyocytes grown under standard culture conditions, which was promoted by the cellular redox unbalance [24] and SFN mitigates male reproductive dysfunction induced by a high-fat diet through the inhibition of ER stress and apoptosis [19]. The administration of SFN can enhance embryonic angiogenesis that is suppressed by ethanol, primarily by reducing excessive ROS production and alleviating ER stress [27]. Factors such as the severity and duration of stress, the specific cell type, and the presence of other stressors can influence whether ER stress leads to cell survival or death [17]. In summary, we can conclude that the impact of ER stress on cell fate is highly context-dependent. The dual nature of ER stress highlights its complexity in cellular processes. While our findings support the role of SFN in increasing ER stress and promoting apoptosis in GBM cells, it is important to consider the broader context and potential protective mechanisms that may also be at play. Further research is needed to fully understand these dynamics and to develop targeted therapies that can harness the beneficial aspects of ER stress while mitigating its harmful effects.

## Conclusion

In conclusion, graphical abstract illustrates that SFN treatment markedly enhances ER stress in both glioma cell lines and primary glioma cells, leading to apoptosis. The use of the classic ER stress inhibitor 4PBA partially mitigated the cytotoxic effects of SFN on glioma cells, coinciding with decreased levels of cleaved caspase-3. These findings suggest that SFN could be a promising candidate for targeted therapies in GBM treatment.

## Electronic supplementary material

Below is the link to the electronic supplementary material.

Supplementary Material 1

Supplementary Material 2

## Data Availability

The datasets generated and/or analysed during the current study have been deposited to the NCBI Sequence Read Archive (SRA) under accession number PRJNA1263655.

## Abbreviations

GBM: Glioblastoma
SFN: Sulforaphane
ERS: Endoplasmic reticulum stress
UPR: Unfolded protein response
TM: Tunicamycin
4-PBA: 4-phenylbutyrate
ATF4: Activating transcription factor 4
CHOP: C/EBP homologous protein
XBP1s: Spliced X-box binding protein 1
HA: Human astrocyte
WB: Western blot
IHC: Immunohistochemistry
RNA-seq: RNA sequencing
qPCR: Quantitative polymerase chain reaction
