# Supplementary material for: Sulforaphane induces cell morphology change and cell apoptosis by activating endoplasmic reticulum stress in glioblastoma
Source: BMC Cancer. 2025 Jul 1;25:1050. doi: 10.1186/s12885-025-14378-4 (PMC12210932; doi:10.1186/s12885-025-14378-4)

Full uncropped Gels and Blots image(s)

Full uncropped images of GRP79

A

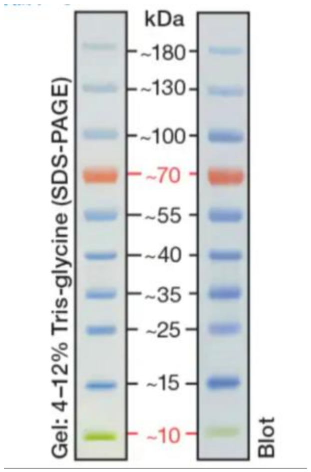

B

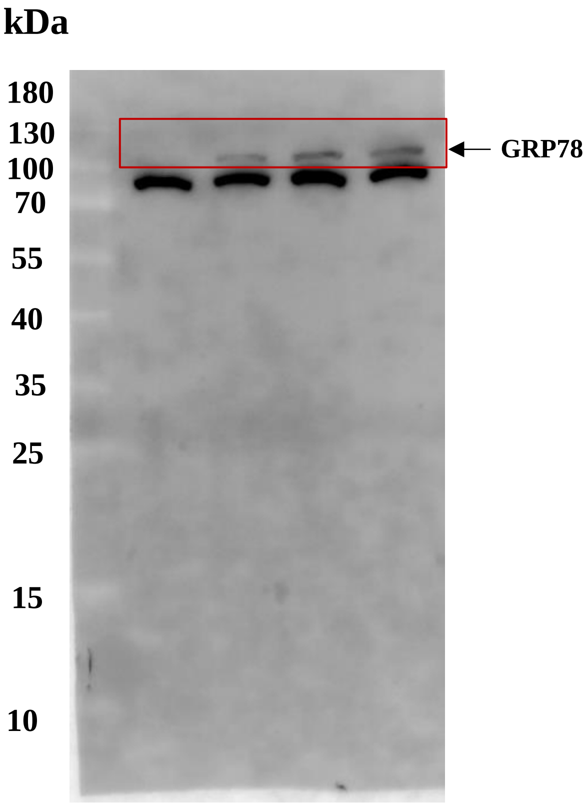

C

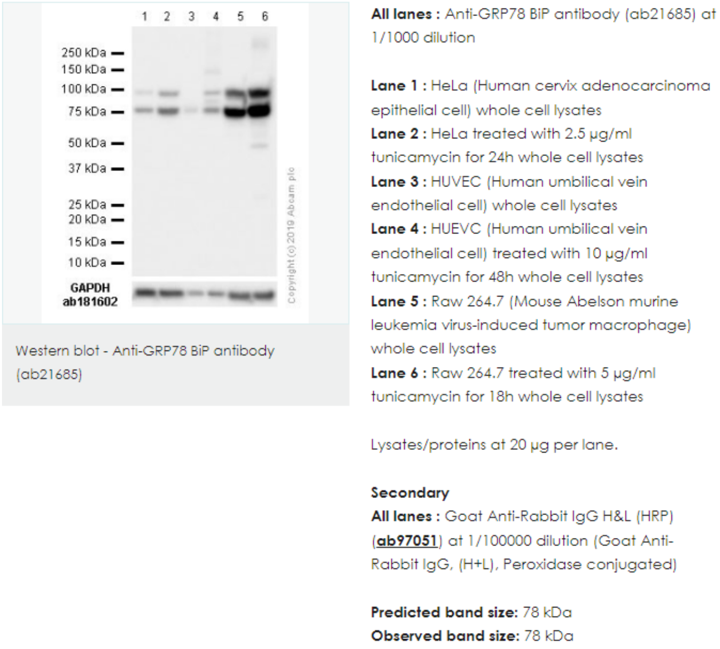

D

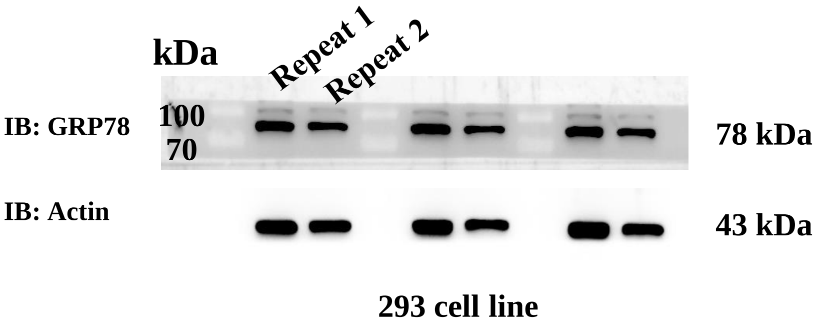

Full uncropped images of p-eIf2α and ATF6

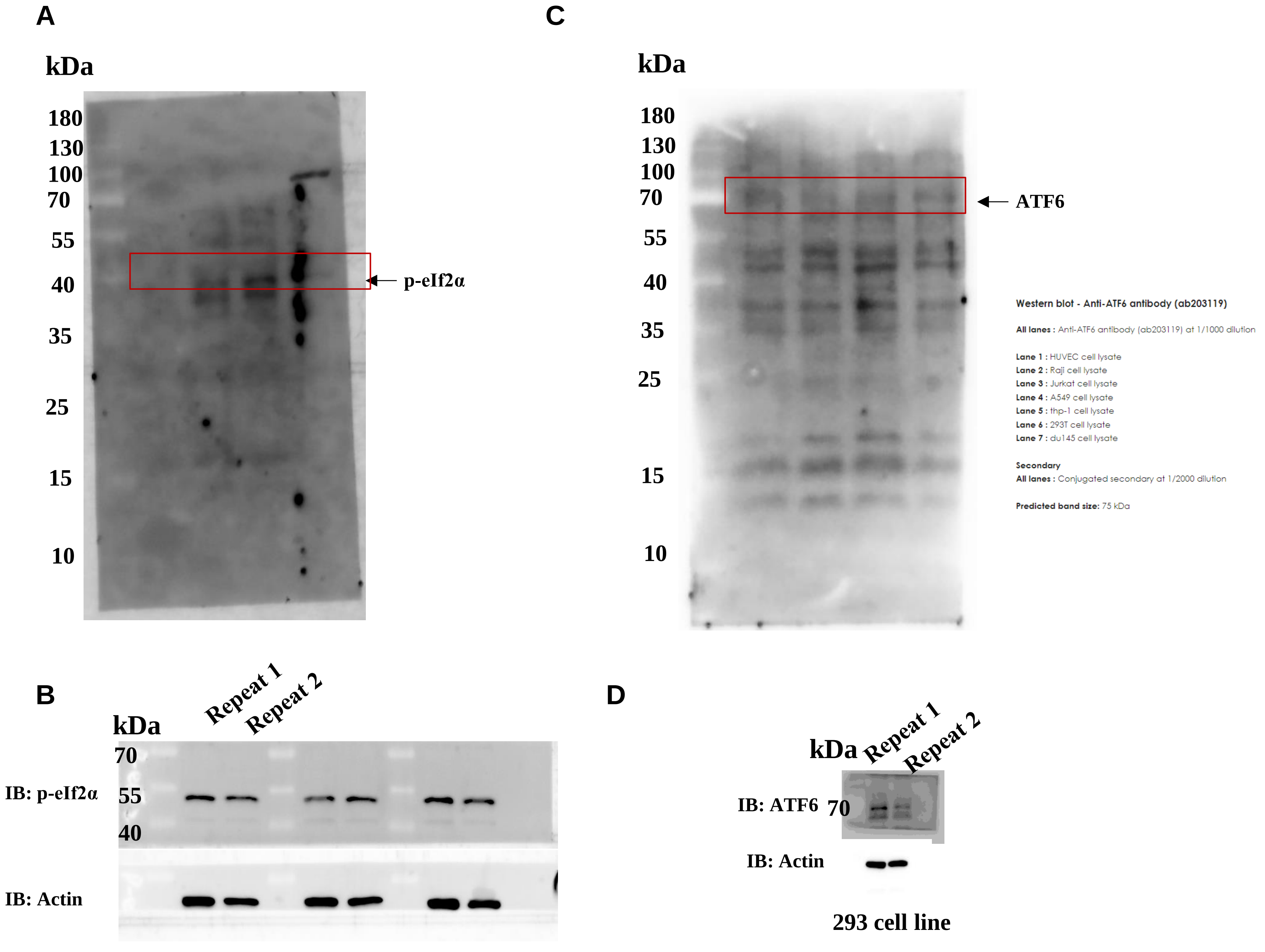

Full uncropped images of XBP1s and ATF4

A

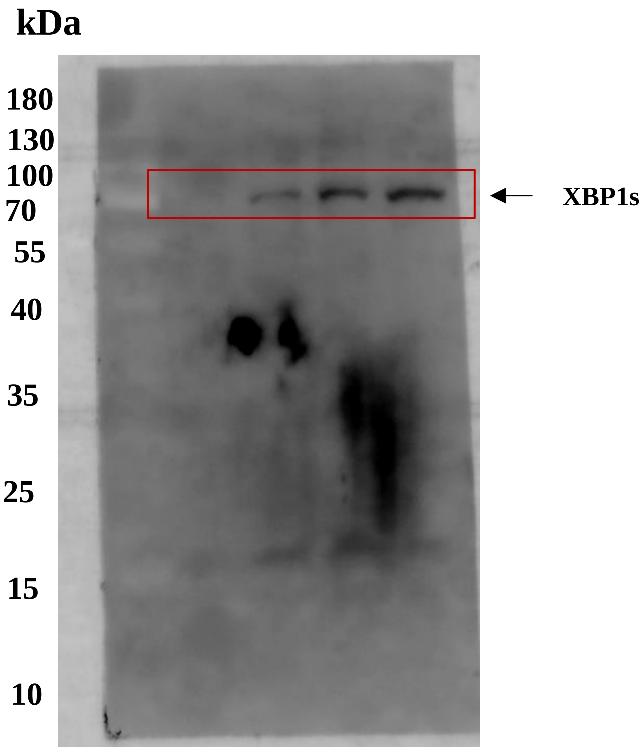

C

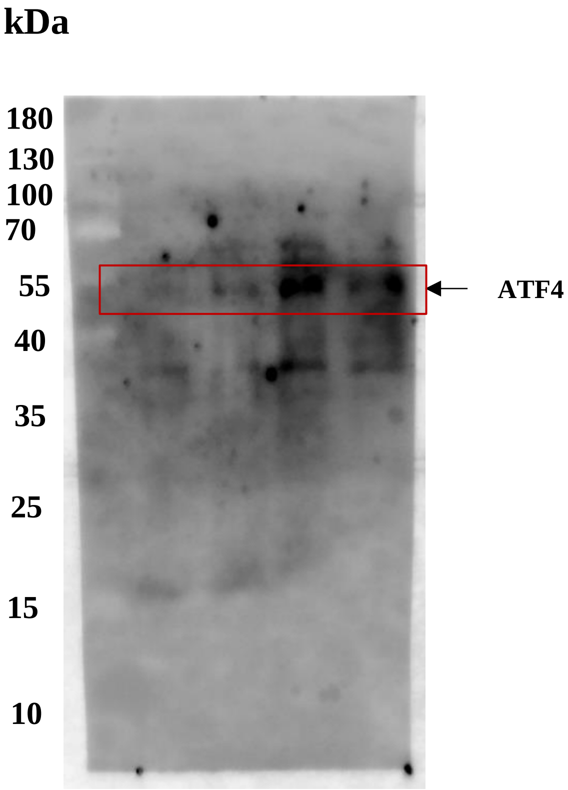

B

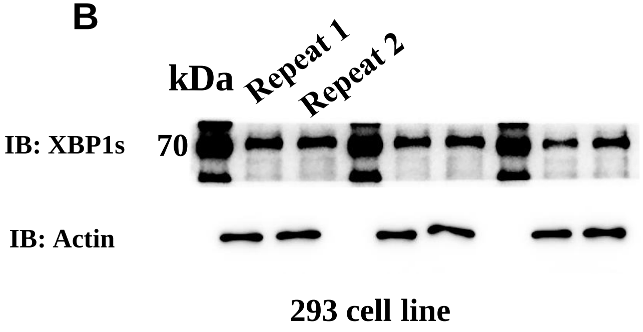

D

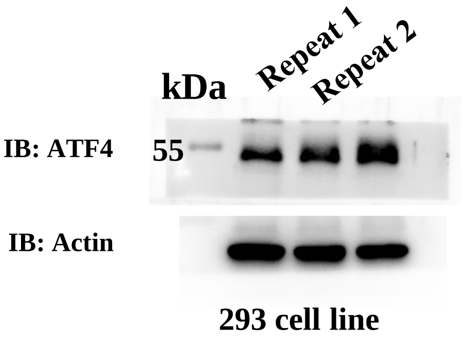

Full uncropped images of cleaved-Caspase3 and CHOP

A

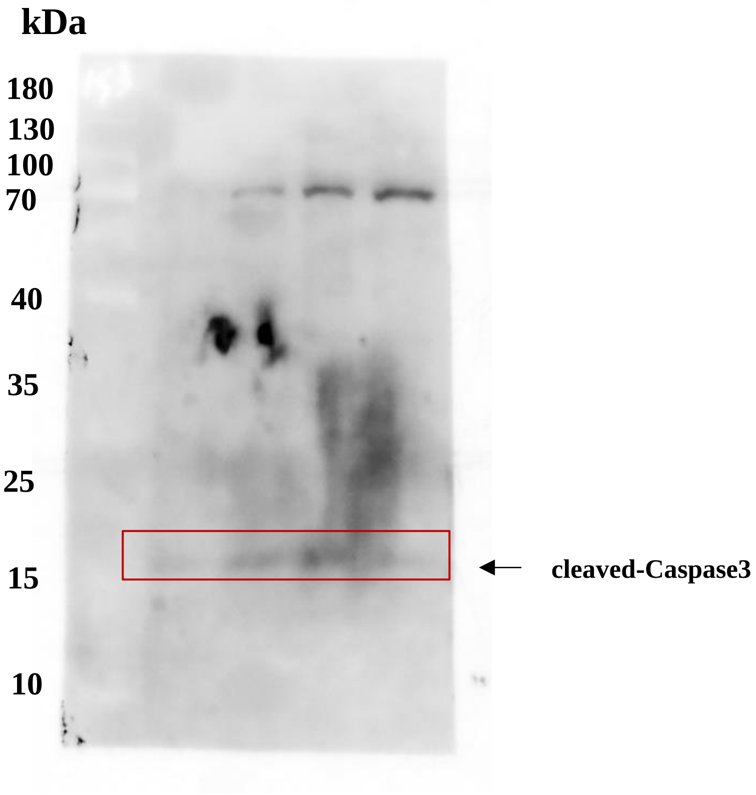

B

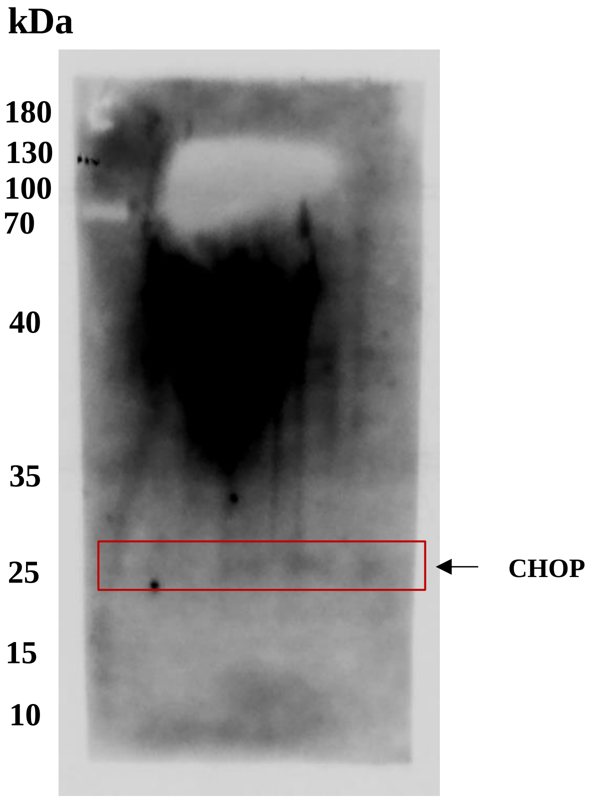

Raw Blot images in Fig. 2D

2D-1

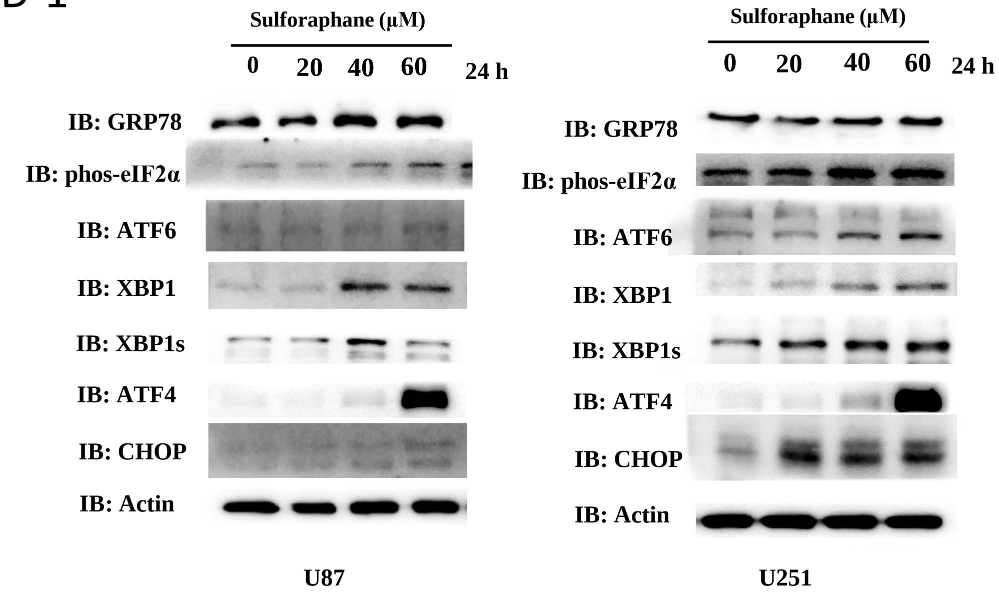

2D-2

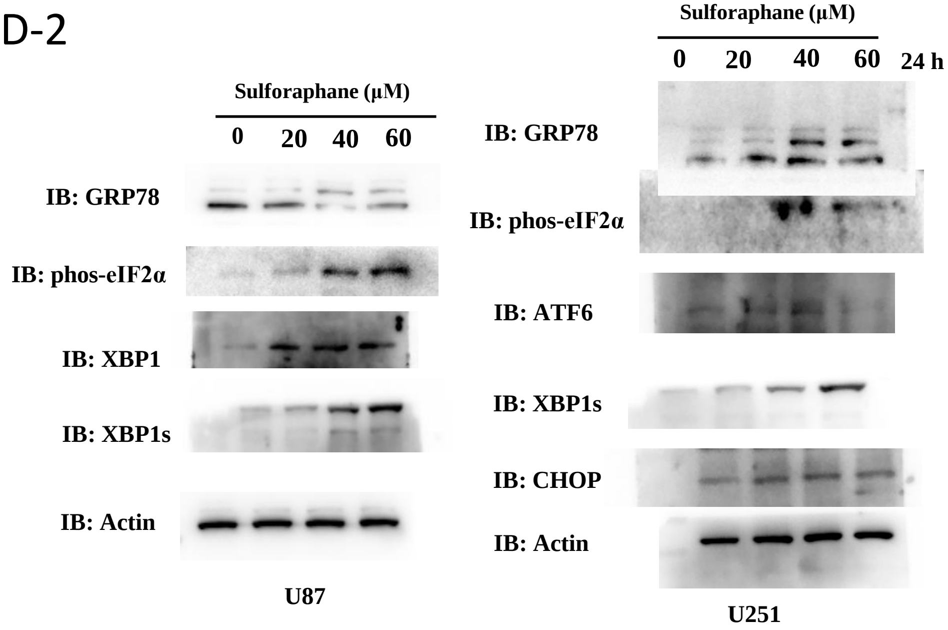

2D-3

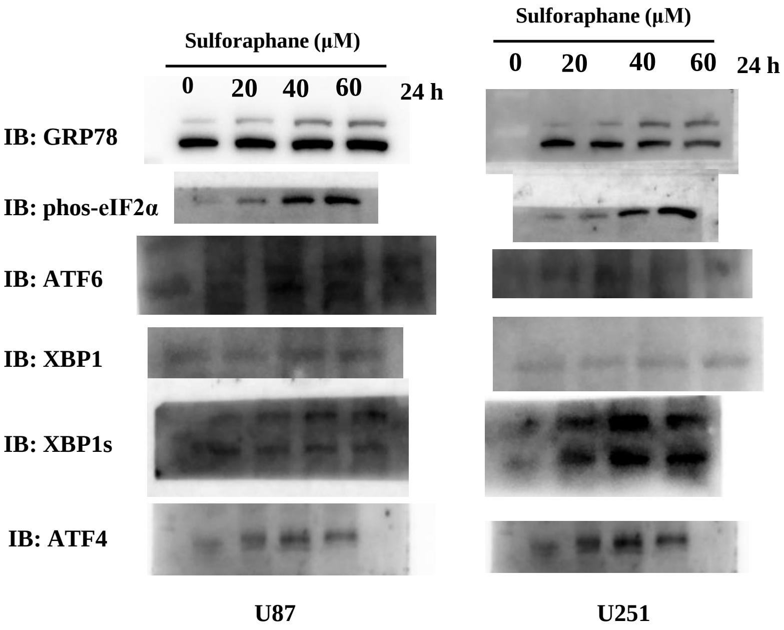

Raw Blot images in Fig. 3A

3A-1

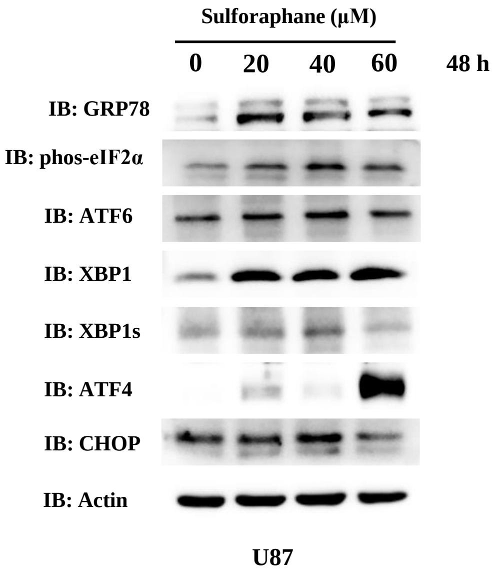

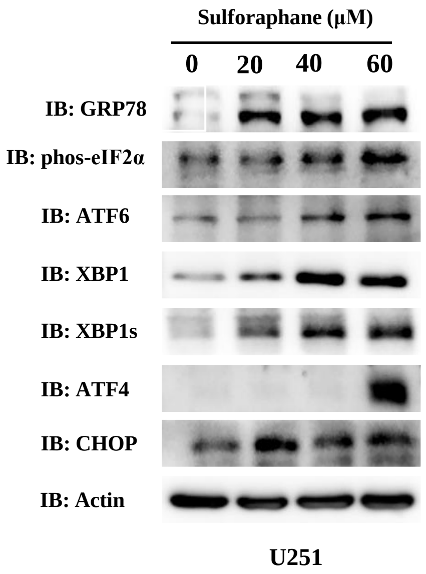

3A-2

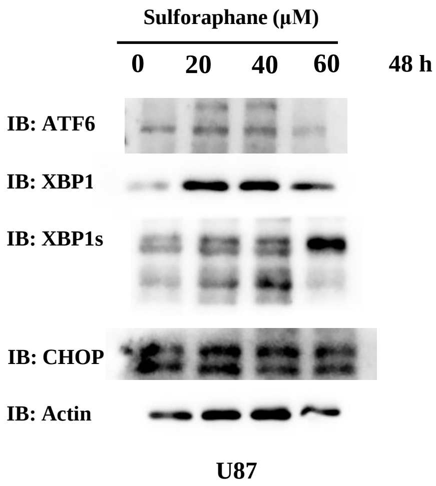

3A-3

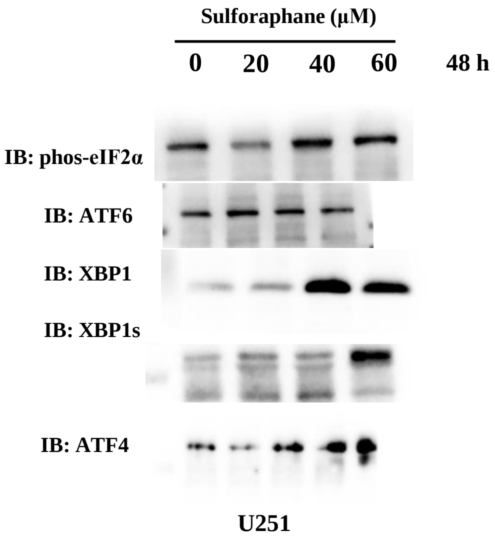

Raw Blot images in Fig. 4B

4B-1

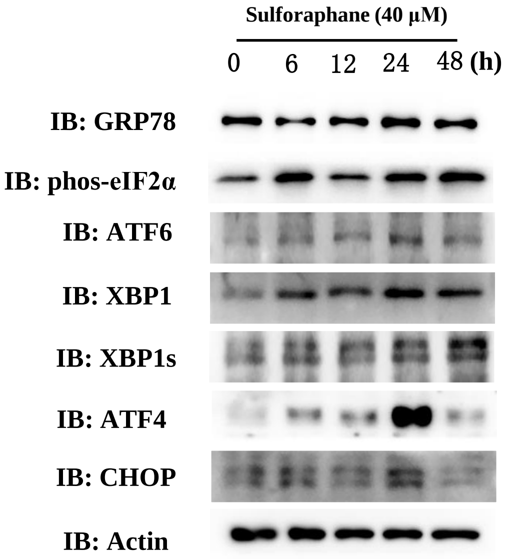

4B-2

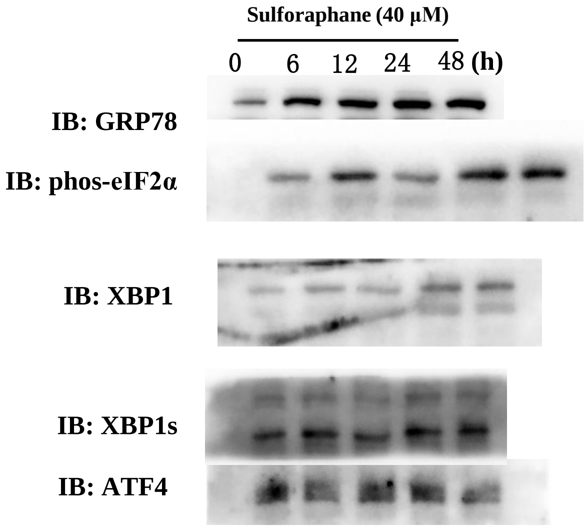

4B-3

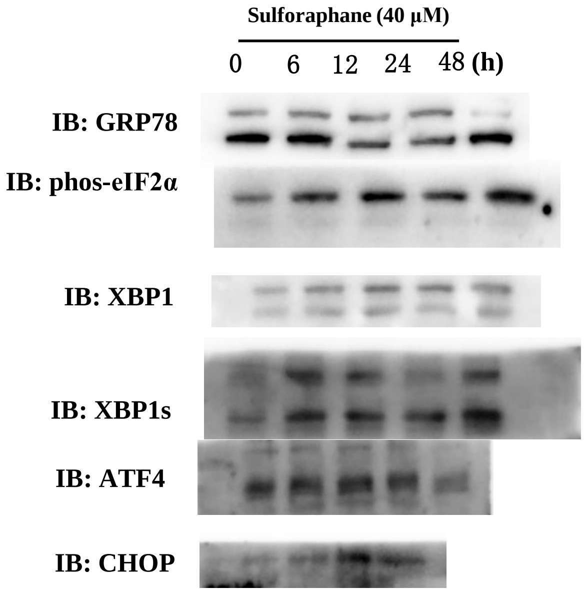

Raw Blot images in Fig. 4E

4E-1

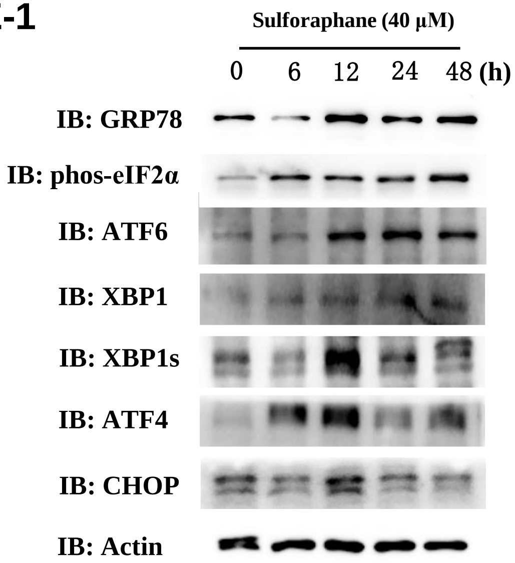

4E-2

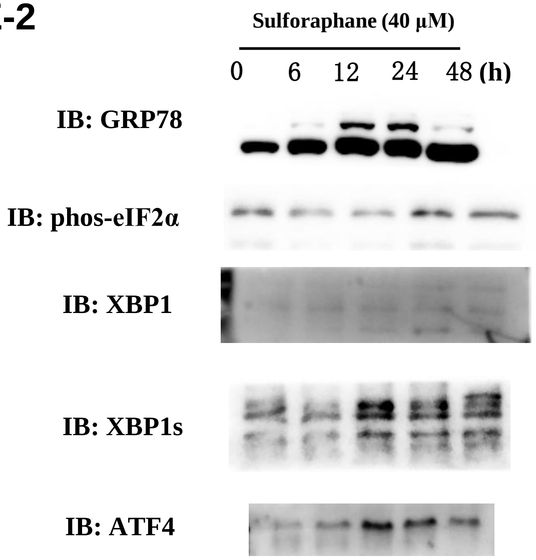

4E-3

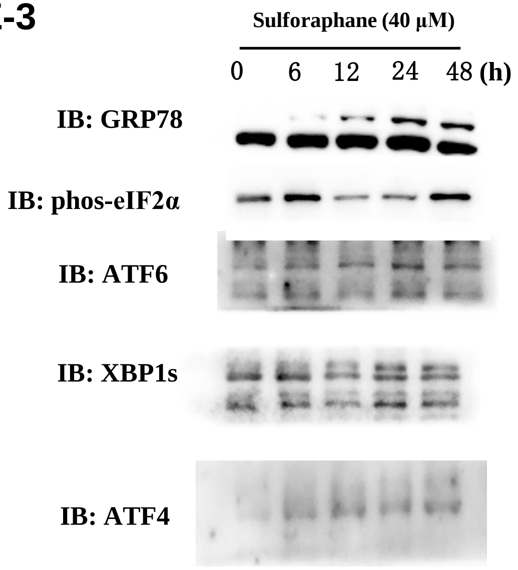

Raw Blot images in Fig. 6C

6C-1

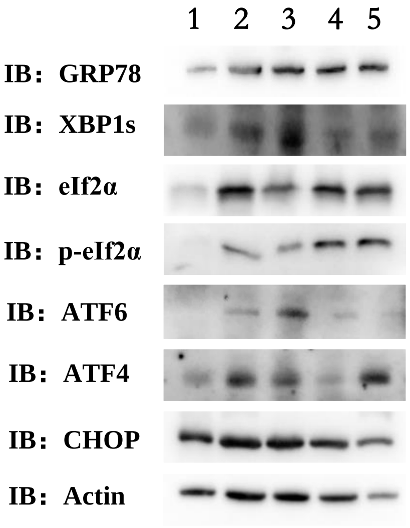

6C-2

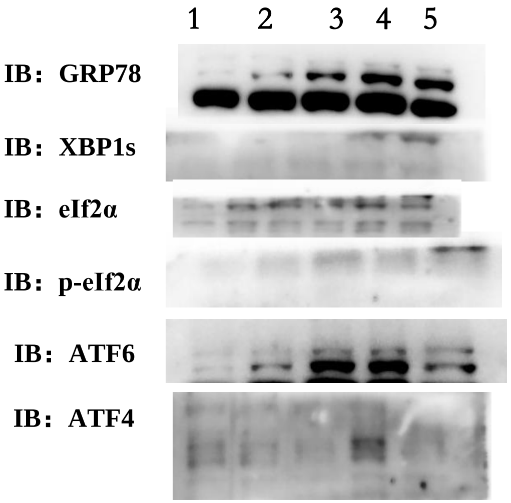

6C-3

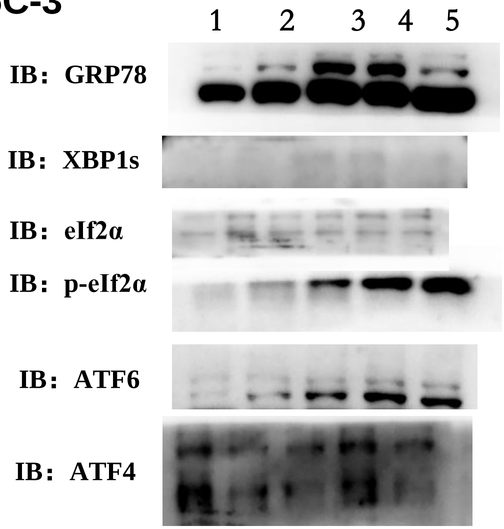

Raw Blot images in Fig. 6E

6E-1

DMSO	+	-	-	-	-	-
SFN	-	+	+	-	-	-
TM	-	-	-	-	+	+
4-PBA	-	-	+	+	-	+

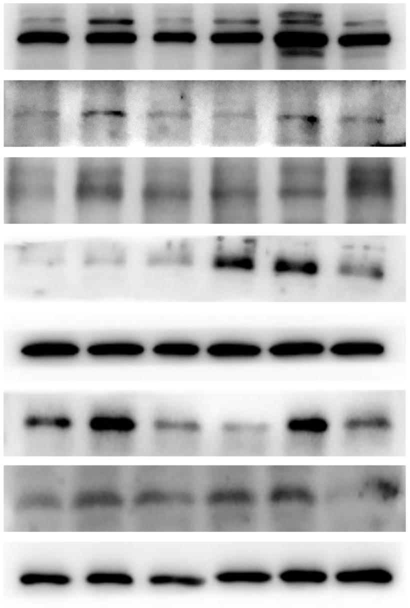

6E-2

DMSO	+	-	-	-	-	-
SFN	-	+	+	-	-	-
TM	-	-	-	-	+	+
4-PBA	-	-	+	+	-	+

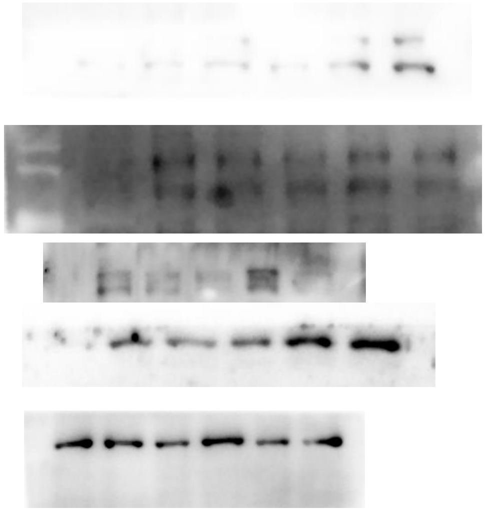

6E-3

DMSO	+	-	-	-	-	-
SFN	-	+	+	-	-	-
TM	-	-	-	-	+	+
4-PBA	-	-	+	+	-	+

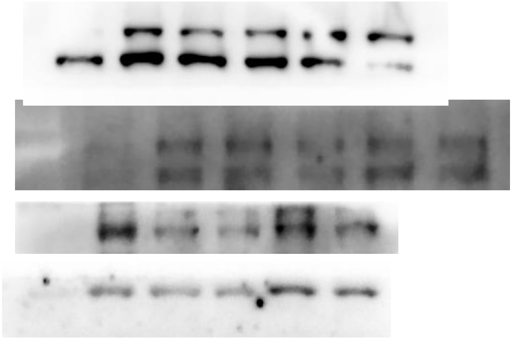

Supplement: Supplementary file 1 — Supplementary Material 1 [file 12885_2025_14378_MOESM1_ESM.pdf]
