# Supplementary material for: Sulforaphane induces cell morphology change and cell apoptosis by activating endoplasmic reticulum stress in glioblastoma
Source: BMC Cancer. 2025 Jul 1;25:1050. doi: 10.1186/s12885-025-14378-4 (PMC12210932; doi:10.1186/s12885-025-14378-4)
Supplement: Supplementary file 2 — Supplementary Material 2 [file 12885_2025_14378_MOESM2_ESM.docx]

Supplementary table S1. Information on patients with gliomas of different grades.

| **Samples Number** | **Age** | **Sex** | **Grades** |
| --- | --- | --- | --- |
| 1 | 32 | Female | I |
| 2 | 59 | Female | II |
| 3 | 40 | Female | III |
| 4 | 56 | Female | IV |
| 5 | 65 | Male | IV |

The age, pathological staging, and other relevant conditions of five glioma patients.

Supplementary table S2. Primers sequences used in this study.

| **Primer** | **Forward** | **Reverse** |
| --- | --- | --- |
| GAPDH | CCACTCCTCCACCTTTG | CACCACCCTGTTGCTGT |
| GRP78 | GCACAGACGGGTCATTCCAC | TCCTATGTCGCCTTCACTCC |
| CHOP | TCTTCCTCCTCTTCCTCCTG | CACTCTTGACCCTGCTTCTC |
| XBP1s | GCTGAGTCCGCAGCAGGTGCA | CCCAGCTCCGGAACGAGGTCA |
| ATF4 | GTCCTGTCCTCCACTCCAGA | GGGTGTCTTCCTCCTTTATGC |
| PERK | CTGGTTCTTTGGTTGCTTGG | GCCCACTTTCACCTTCAGAG |
| IRE1 | AGCCACTACCCGAAGGTGAG | TTCAGGCTCTGGGAACACAT |
| elF-2α | AGGACTGCCTGGGTCTTTG | CTTCCCGTTCATCTTCATTCA |

The primer sequence information used in the article.

Supplementary table S3. Antibodies used in this study for Western Blot and Immunohistochemistry.

| **Antibodies** | **Source** | **Identifier** |
| --- | --- | --- |
| Rabbit anti-GRP78 | abcam | Cat. # ab21685 |
| Rabbit anti-p-eIF2α | Cell Signaling Technology | Cat. # 3398 |
| Rabbit anti-ATF6 | abcam | Cat. # ab203119 |
| Rabbit anti-XBP1 | abcam | Cat. # ab37152 |
| Rabbit anti-XBP1s | Proteintech | Cat. # 24868-1-AP |
| Rabbit anti-ATF4 | Cell Signaling Technology | Cat. # 11815 |
| anti-CHOP | Santa Cruz Biotechnology | Cat. #sc-7351 |
| anti-Actin | Affinity Biosciences | Cat. #T0022 |

A summary of the antibody information used in the article.


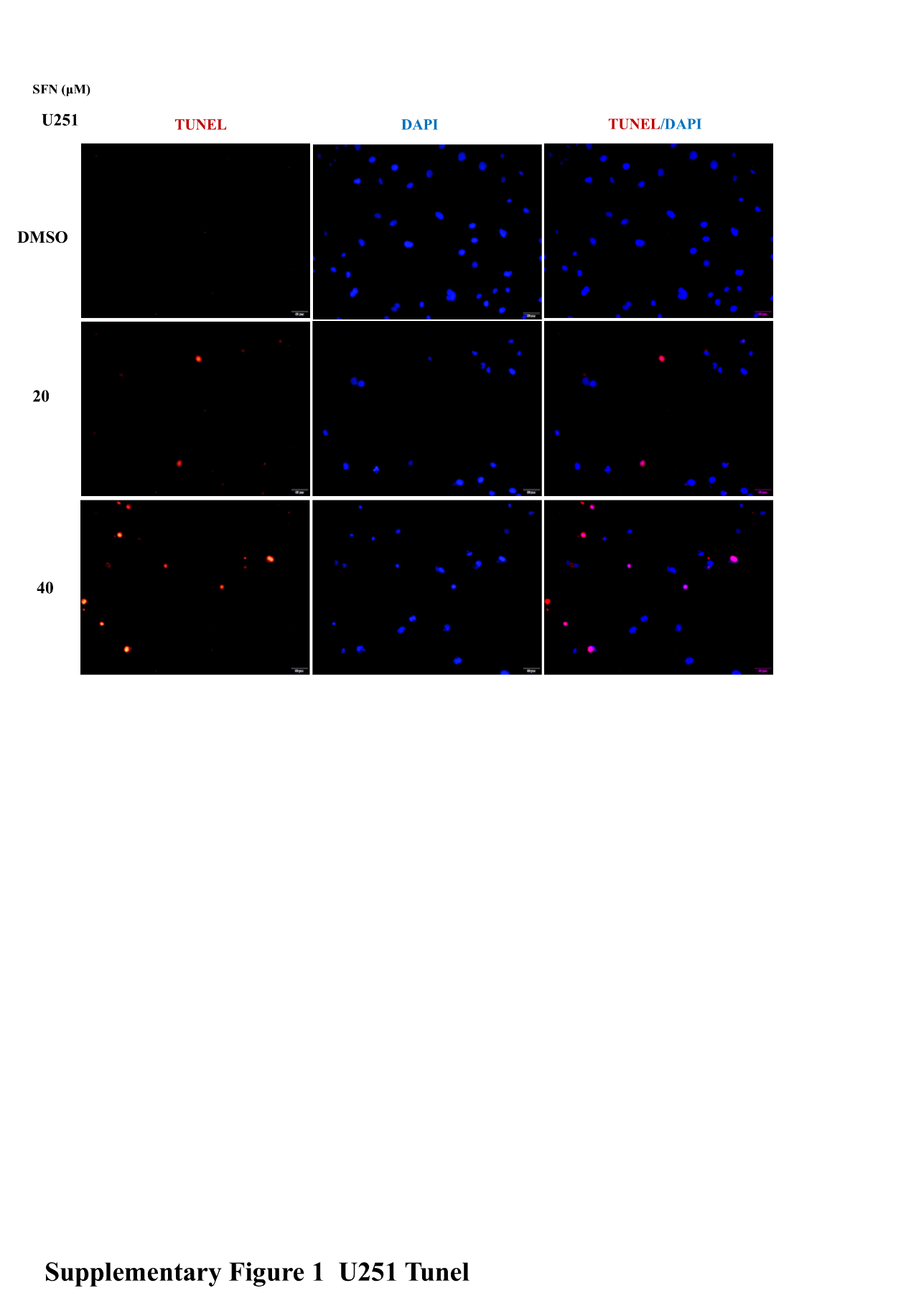


**Supplementary Fig.1 SFN Promotes Cell Apoptosis in U251 Cells.**

TUNEL staining of U251 cells treated with SFN at the indicated concentrations for 24 h.


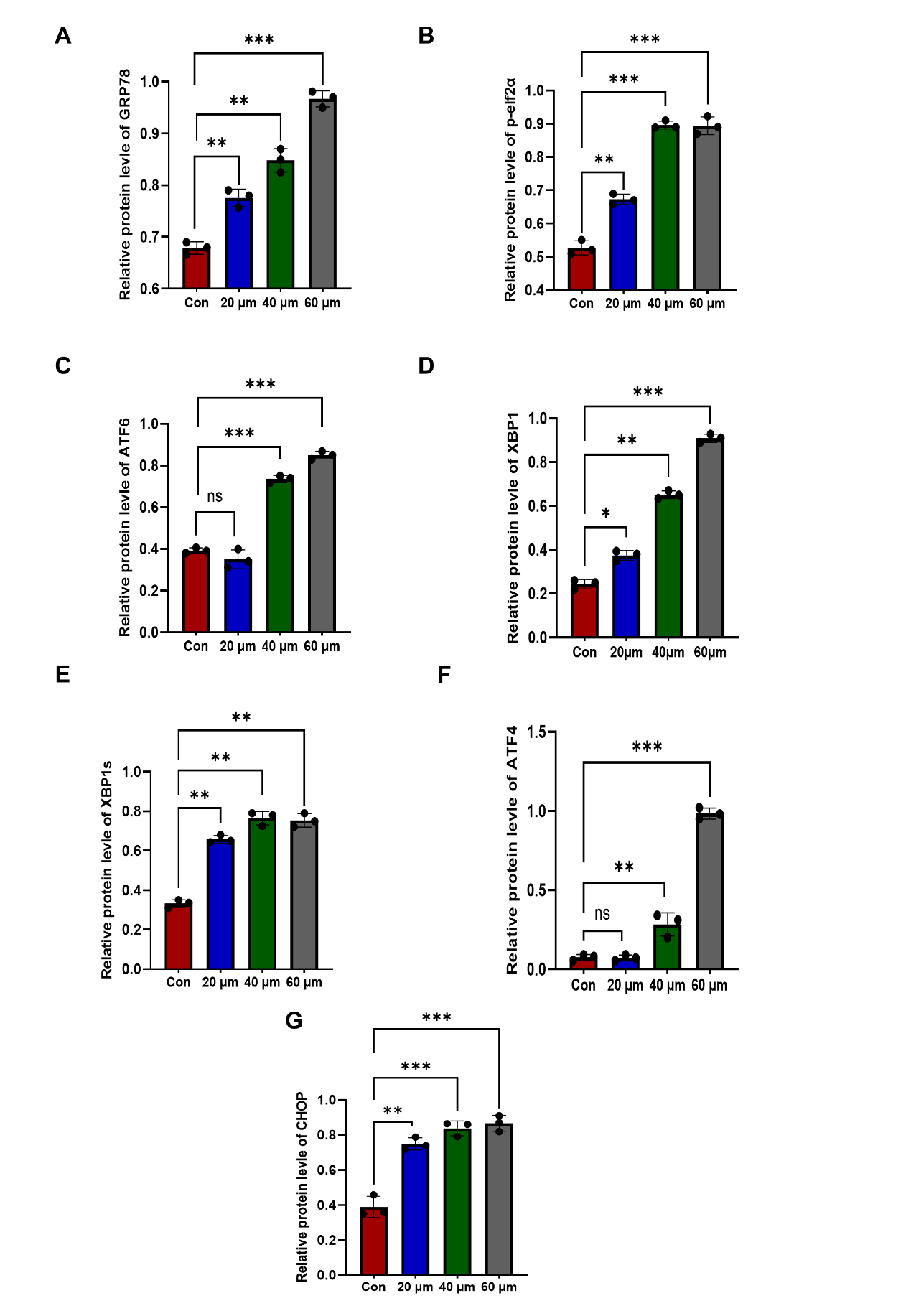


**Supplementary Fig.2 Enhanced ER Stress Response with Increased Stimulation Duration of SFN in U251 Cells.**

Quantitative analysis of protein levels of GRP78 (A), p-eIf2α (B), ATF6 (C), xBP1 (D), xBP1s (E), ATF4 (F) and CHOP (G) in A right U251 cells.


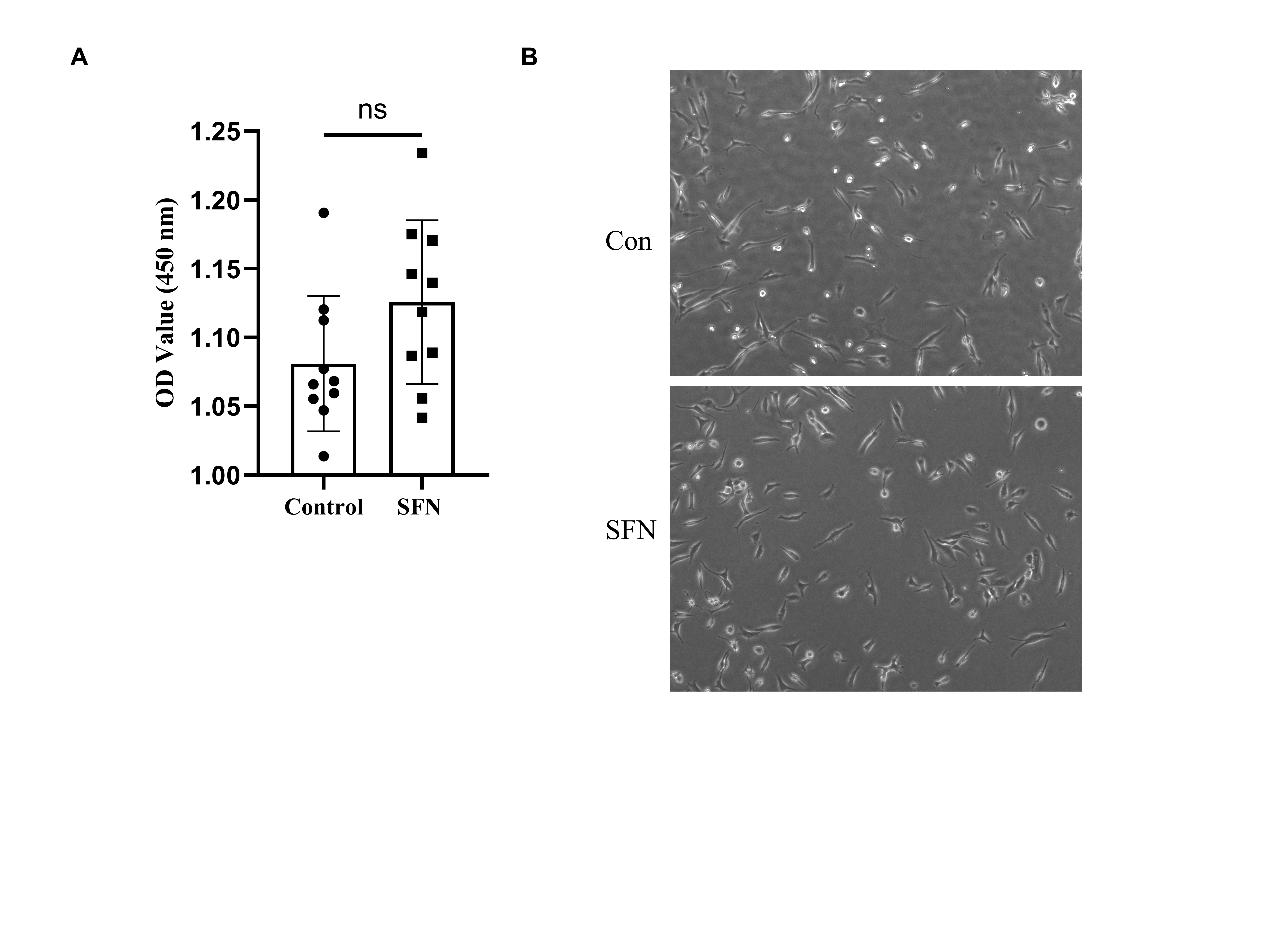


**Supplementary Fig.3 Assessment of SFN cytotoxicity and selectivity in normal human astrocytes (HA cells).**CCK-8 assay was used to evaluate cell viability of HA cells treated with SFN (60 μM). SFN treatment did not significantly reduce HA cell viability, suggesting minimal cytotoxicity toward normal astrocytes (A). Morphological examination of HA cells by light microscopy revealed no significant changes in cell shape or density following SFN treatment, further supporting the selective anti-tumor activity of SFN in glioblastoma cells (B).
